# Sequencing Strategies to Guide Decision Making in Cancer Treatment

**DOI:** 10.1371/journal.pmed.1002189

**Published:** 2016-12-06

**Authors:** James T. Topham, Marco A. Marra

**Affiliations:** 1 Canada’s Michael Smith Genome Sciences Centre, BC Cancer Agency, Vancouver, Canada; 2 Department of Medical Genetics, University of British Columbia, Vancouver, Canada

## Abstract

In a Perspective, James Topham and Marco Marra discuss progress in the use of genomic information to guide cancer treatment.

Cancer is a complex genetic disease often associated with the accumulation of somatic DNA alterations [1]. Genes that are targets of somatic alteration in cancer can be broadly classified as tumor suppressors or oncogenes, depending on whether they bear loss-of-function or gain-of-function alterations, respectively [2]. Comprehensive efforts into cataloguing cancer genes have revealed that tumors demonstrate substantial variability in the genes that accumulate mutations both within and across cancer types [3,4]. Targeted agents have been developed to directly target tumors by acting on specific genomic alterations or gene products that are unique to the tumor cells, thereby offering a complementary approach to broad-acting cytotoxic chemotherapies.

Targeted treatments have become a major modality in the treatment of cancer and can offer advantages to the patient over standard chemotherapy alone [5,6]. However, given the heterogeneity of cancer, appropriate selection of targeted drugs relies on our ability to identify actionable genomic alterations within individual tumors [7]. Rapid advances in genomic technologies have fueled translational cancer research, providing opportunities for acquisition of precision genomic information to help guide clinical management of cancer patients. Concomitant with decreasing costs, the advancement of genomic technologies has placed clinicians and researchers in a unique position to translate genomic results to the clinic.

The first assays to screen cancer patients for somatic alterations targetable by drugs were based on detection of single gene alterations [8]. For example, early studies investigating the genomic landscapes of breast cancers identified *HER2* copy number amplifications in ~30% of patients, noting that overexpression of *HER2* was associated with poor prognosis [9]. Trastuzumab was among the first monoclonal antibody-based targeted drugs developed, targeting *HER2*-expressing tumor cells. The success of clinical trials investigating the use of trastuzumab in the treatment of *HER2*-positive breast cancers has resulted in routine screening for *HER2* amplifications in the management of breast cancer patients [10]. Another early example of a successful single-gene assay is that of chronic myeloid leukemia (CML), in which the BCR–ABL fusion protein results from a chromosomal translocation event found in ~90% of CML patients [11]. Imatinib, a tyrosine kinase inhibitor developed in the late 1990s, exhibited significant antitumor effects in cells harboring the *BCR–ABL* fusion [12]. Screening for the presence of *BCR–ABL* fusion transcripts thus became routine in the use of imatinib for CML patients [13].

Continued discovery of recurrent driver alterations in different cancers, concomitant with further development of targeted drugs, eventually generated an impetus for the construction of multiplexed assays to detect multiple genomic alterations in cancer patients. Extending from single-gene-based assays, panel sequencing offered the opportunity to discover genomic alterations in tumors that were not known to harbor such alterations. Early applications of multi-gene assays aimed at guiding cancer treatment focused on a small number of genes directly associated with targeted agents, including examples such as a panel capable of detecting 41 alterations in 9 genes relevant in lung cancer [14], as well as a panel used to detect 120 mutations in 13 genes relevant across a broad spectrum of tumor types [15]. Advancements in genomic technology have since led to development of panels encompassing as many as 4,000 alterations across 143 genes [16], and incorporation of panel methods has become routine in several cancer control organizations such as the BC Cancer Agency [17] and Memorial Sloan Kettering Cancer Center [18].

Investigations into the feasibility and efficacy of applying panel sequencing techniques to large populations of patients with advanced cancer are ongoing and include trials such as NCI-MATCH (Molecular Analysis for Therapy Choice) [19], TAPUR (Targeted Agent and Profiling Utilization Registry) [20], and TOP (The Oncopanel Pilot; clinicaltrials.gov/show/NCT02171286). Parallel to these efforts is the recently completed SHIVA trial, which compared clinical outcomes between patients receiving targeted agents, selected based on panel sequencing, and those given conventional chemotherapy, in patients with any type of metastatic solid tumor refractory to standard treatment [21]. Results of SHIVA showed no or limited improvement in progression-free survival for patients receiving targeted therapy [22]. However, it remains unclear whether these results from the SHIVA study can be attributed to an inability of panel sequencing to guide effective therapy rather than other factors, such as the selection of genes included in the panel or a restricted selection of targeted agents from which to choose.

While offering the potential to detect a broader range of actionable genomic alterations compared to single-gene assays, panel sequencing technologies generally do not survey all informative or actionable alterations. The obvious extension to panel-based methods are whole-genome sequencing (WGS) approaches, which are now being explored and offer the substantial advantage of comprehensive detection of genomic alterations that elude detection using panel sequencing. Among the first demonstrations of the use of WGS technology to guide treatment decision-making in cancer was a case of late-stage, metastatic adenocarcinoma of the tongue [23]. Integration of whole-genome and transcriptome data provided a rationale for the selection of a targeted agent that would not have been otherwise considered and, in this case, was associated with maintenance of stable disease for several months [23]. The application of WGS approaches in guiding personalized treatment of cancer has since been expanded to a broader patient population in the POG (Personalized OncoGenomics; clinicaltrials.gov/ct2/show/NCT02155621) trial, with preliminary results suggesting that WGS technology can inform treatment in ~34% of late-stage cancer patients with metastatic diseases [24]. Whether WGS technology has the potential to guide treatment selection in a substantial proportion of cancer patients or to improve treatment outcomes remains unclear and may depend on the continued identification of driver alterations, development of additional targeted drugs to select from, broader clinical indications for existing targeted drugs, technology innovations that speed turnaround times and cost reductions, and development of clinical study designs able to rigorously evaluate treatment success.

Complicating the application of precision approaches to guide cancer treatment are results from detailed investigations into the clonal landscapes of tumors, which have revealed the dynamic nature of cancers resulting from evolution in response to selective pressures [25]. Consequences of the inherent plasticity of tumors are witnessed in the high frequency of relapse for many cancers and drive the need to cease treating the disease as a static entity. The emergence of an evolutionary approach to cancer therapy is currently taking place. For example, the TRACERx (Tracking Cancer Evolution Through Treatment; clinicaltrials.gov/ct2/show/NCT01888601) trial follows lung cancer patients to assess the influence of therapies on the evolution of cancer over time [26]. Thus, while sequencing technologies have clear potential to inform treatment decisions, the proportion of cases benefiting from such approaches will perhaps be maximized when strategies addressing the dynamic nature of tumors are implemented. Meanwhile, further advances in technology will continue to narrow the gap between genomics applications and the guidance of treatment planning in cancer, potentially offering benefit to patients, clinicians, and health systems.

## Abbreviations

CML: chronic myeloid leukemia
WGS: whole-genome sequencing
